# Gut microbiota dysbiosis in infants and young children with severe pneumonia and sepsis: a matched case-control study identifying potential biomarkers for early risk stratification

**DOI:** 10.3389/fimmu.2026.1796938

**Published:** 2026-04-22

**Authors:** Cheng Yang, Peijin He, Yang Wen, Qin Zeng, Jiawei Li

**Affiliations:** 1 Department of Pediatric Intensive Care Unit Nursing, West China Second University Hospital, Sichuan University, Chengdu, China; 2 Department of Pediatric Intensive Care Unit Nursing, WCSUH-Tianfu·Sichuan Provincial Children’s Hospital, Meishan, China; 3 Key Laboratory of Birth Defects and Related Diseases of Women and Children (Sichuan University), Ministry of Education, Chengdu, China; 4 Department of Gastroenterology Nursing, West China Second University Hospital, Sichuan University, Chengdu, China; 5 Department of Pediatric Outpatient Nursing, West China Second University Hospital, Sichuan University, Chengdu, China

**Keywords:** biomarker, dysbiosis, gut microbiota, infants, pediatric intensive care, sepsis, severe pneumonia

## Abstract

**Background:**

Sepsis remains a life-threatening complication of severe pneumonia in infants and young children, yet early biomarkers are lacking. The gut microbiota modulates host immunity, but the association between the gut microbiota and pediatric pneumonia-associated sepsis is unclear due to confounding factors.

**Methods:**

In this prospective, 1:1 matched case-control study, we enrolled 100 infants and young children (28 days–36 months) with severe pneumonia, stratifying them into sepsis (n=50) and non-sepsis (n=50) groups matched for age and antibiotic exposure. Fecal samples collected within 48 hours of PICU admission underwent 16S rRNA gene sequencing. Diversity, taxonomic composition, and differential taxa were analyzed.

**Results:**

The sepsis group exhibited significantly reduced alpha diversity (Shannon index: 2.30 ± 1.50 vs. 2.83 ± 1.36, P = 0.027), increased Enterobacteriaceae (18.97% vs. 9.44%, P = 0.046), and decreased Lachnospiraceae (2.01% vs. 8.11%, P = 0.010). LEfSe (Linear discriminant analysis Effect Size) further revealed distinct microbial signatures: the sepsis group exhibited enrichment of Lactobacillaceae and Clostridium butyricum, while the non-sepsis group was characterized by higher abundance of Lachnospiraceae and Segatella.

**Conclusion:**

Sepsis in infants and young children with severe pneumonia is associated with a specific gut microbiota signature, independent of major confounders. This dysbiotic profile, involving taxa associated with endotoxin production and short-chain fatty acid metabolism, may serve as an early biomarker for risk stratification and could inform microbiota-targeted interventions in critically ill infants and young children.

## 1 Introduction

Sepsis is one of the leading causes of death among children worldwide, posing an especially severe threat to infants and young children. Severe pneumonia is the most common trigger of sepsis in this population, and the co-occurrence of both conditions significantly increases therapeutic difficulty and mortality risk (1, 2). Despite advances in anti-infective therapies and life-support technologies, there remains a lack of sensitive and specific biomarkers for the early identification and prognostic assessment of sepsis, and many aspects of the pathophysiology of sepsis are still not fully understood (3, 4).

In recent years, the gut microbiota has been recognized as an important “microecological organ” associated with the onset and progression of sepsis, owing to its central role in immune regulation, metabolism, and barrier maintenance (5, 6). Studies in adults have shown that sepsis patients often exhibit significant gut dysbiosis, characterized by reduced diversity, expansion of opportunistic pathogens, and depletion of beneficial bacteria, with such disturbances closely linked to the degree of inflammation, organ failure, and clinical outcomes (7, 8). However, infants and young children are not simply “small adults”—their gut microbiota is in a critical period of rapid development and colonization, displaying unique compositional structure, functional vulnerability, and dynamism (9, 10). Their immature immune systems and relatively compromised intestinal barrier function may be associated with a higher susceptibility to dysbiosis, with potentially more severe consequences. Therefore, extrapolating findings from adult studies to pediatric populations has limitations, underscoring the necessity for age-specific investigations (9).

Although a limited number of studies have focused on the gut microbiota of critically ill children, notable research gaps persist. First, most studies have not rigorously distinguished between severe infection alone and secondary sepsis, making it difficult to determine whether observed microbial alterations are attributable to the infection itself or to sepsis-specific pathophysiological processes (1, 7). Second, pediatric studies often fail to adequately control for major confounders influencing the microbiota, such as antibiotic exposure and feeding patterns, which limits the interpretability and generalizability of the results (9, 11). Finally, there is still insufficient evidence from well-matched clinical studies regarding which microbial features may hold diagnostic or prognostic predictive potential for sepsis in infants and young children (4).

Therefore, this study aimed to directly compare the gut microbial structure between infants and young children with severe pneumonia complicated by sepsis and those with severe pneumonia alone, using a prospective, meticulously matched case-control design. We strictly controlled for key confounders including age and antibiotic exposure, focusing specifically on the microbial alterations that characterize sepsis itself in this clinical context (12, 13). This research not only helps to characterize the dysbiotic patterns associated with sepsis in early life and understand their underlying pathophysiological mechanisms, but also aims to provide a scientific basis for the early identification of high-risk patients and the development of age-specific, microbiota-informed diagnostic or interventional strategies (5, 14). Notably, the first 36 months of life represent a critical window for gut microbiota assembly and immune programming, during which the microbial ecosystem undergoes rapid succession and remains highly dynamic (9, 10). Unlike the stable adult microbiome, the developing gut microbiota in this age group exhibits unique compositional features and functional vulnerabilities, making it particularly vulnerable to dysbiosis in the context of critical illness. Therefore, focusing on this ‘developing population’ is essential to understand age-specific pathophysiological mechanisms of sepsis, rather than simply extrapolating findings from adult studies.

## 2 Research methods

### 2.1 Study design

This study employed a prospective, case-control design to compare the characteristics of gut microbiota between infants and young children with severe pneumonia complicated by sepsis and those with severe pneumonia alone. Matching was performed in a 1:1 ratio, prioritizing age in months (± 3 months). Subsequently, cases and controls were matched based on key parameters of antibiotic exposure within one month prior to hospital admission and fecal sampling, including: ① the number of antibiotic classes (e.g., penicillins, cephalosporins, carbapenems) and ② the cumulative duration of antibiotic use (days). This rigorous matching strategy was designed to ensure that the two groups were highly comparable regarding these major confounders, thereby isolating the microbial signature associated with sepsis itself from that driven by antibiotic exposure. The study was conducted in the Pediatric Intensive Care Unit (PICU) of a tertiary women and children’s hospital located in Chengdu, Sichuan Province, China, from January 2024 to December 2024. The study protocol was approved by the hospital’s Medical Ethics Committee (Approval No.: Medical Research Ethics Review 2023, No. 386). Written informed consent was obtained from the legal guardians of all participating children.

### 2.2 Inclusion and exclusion criteria

#### 2.2.1 Case group

(1) Inclusion Criteria:①Age between 28 days and 36 months (infancy and early childhood, a critical developmental window for gut microbiota);

②Meets the diagnostic criteria for severe pneumonia as defined by the “Guidelines for the Diagnosis and Management of Community-Acquired Pneumonia in Children (2019 Edition) (15)”;

③Diagnosis of sepsis: Sepsis was diagnosed according to the Sepsis-3 international consensus, requiring the concurrent presence of both of the following criteria:a) Suspected or confirmed infection: All patients in the case group had a diagnosis of severe pneumonia, which served as the infectious focus. Microbiological evidence of infection was required, defined as the identification of a pathogenic microorganism from blood culture, a qualified lower respiratory tract sputum culture (or bronchoalveolar lavage fluid), or specimens from other normally sterile sites (e.g., pleural fluid, cerebrospinal fluid) within 24-48 hours of admission; b) Infection-related organ dysfunction: This was quantified using the pediatric Sequential Organ Failure Assessment (pSOFA) score. The pSOFA score, which assesses dysfunction across six organ systems (respiratory, coagulation, liver, cardiovascular, neurological, and renal) with each scored from 0 to 4, was calculated using validated pediatric-specific criteria. A total score of ≥ 2 points, based on the worst physiological and laboratory parameters within the first 24 hours of enrollment, was considered indicative of organ dysfunction associated with acute infection, thereby meeting the diagnostic criteria for sepsis (16);

④Has resided in Sichuan Province for an extended period (≥6 months) prior to disease onset;

⑤Informed consent obtained from the legal guardian.

(2) Exclusion Criteria: ① Inability to obtain a naturally passed fresh stool sample within 48 hours after enrollment.

② Presence of any underlying condition that may significantly affect the gut microbiota, including congenital gastrointestinal malformations, inflammatory bowel disease, severe congenital heart disease, chronic liver or renal failure, primary immunodeficiency, malignant tumors, progressive neurological disorders, or inborn errors of metabolism;

③ Received interventions with microbiota-modulating agents (e.g., probiotics, prebiotics, synbiotics) or specific medical purpose formula foods (aimed at modulating flora) within 1 month prior to enrollment.

#### 2.2.2 Control group

(1) Inclusion Criteria: ① Age between 28 days and 36 months;

② Meets the diagnostic criteria for severe pneumonia (same as the case group);

③ Non-septic status: A non-septic status was defined by the simultaneous fulfillment of the following conditions: a) Absence of confirmed pathogenic evidence: No pathogenic microorganisms were identified from blood culture, qualified lower respiratory tract sputum cultures (or bronchoalveolar lavage fluid), or specimens from other normally sterile sites within 24-48 hours of admission; b) Absence of sustained infection-related organ dysfunction: This was defined as a pSOFA score < 2 points, calculated using the worst physiological and laboratory parameters within the first 24 hours of enrollment. Patients with a pSOFA score ≥ 2 points but negative cultures, for whom sepsis was clinically suspected, were excluded from the control group to ensure a “pure” non-septic comparator cohort.;

④ Has resided in Sichuan Province for an extended period (≥6 months) prior to disease onset;

⑤ Informed consent obtained from the legal guardian.

(2) Exclusion Criteria:​Identical to those of the case group.

### 2.3 Sample size estimation

The sample size was calculated using a power analysis for a matched case-control design, with the Shannon index of alpha diversity as the primary continuous outcome. Based on prior microbiome and sepsis studies (7, 17, 18), an effect size represented by a standardized mean difference (SMD) of 0.6 was anticipated. Using a significance level (α) of 0.05 (two-tailed) and a statistical power (1-β) of 80%, the initial calculation with the formula n = 16/SMD² indicated a requirement of 45 subjects per group. Accounting for an estimated 15% attrition rate, a final sample size of 53 participants per group (106 total) was targeted for recruitment.

### 2.4 Data collection

Within 48 hours after admission, the first spontaneously passed fresh stool sample (approximately 3 grams) was collected from each enrolled child. The sample was immediately placed into a specific fecal collection tube containing nucleic acid stabilizing solution, thoroughly mixed, and stored at -80 °C in an ultra-low temperature freezer until DNA extraction. This sampling time point was chosen to capture the gut microbial profile during the acute phase of sepsis while minimizing interference from prolonged ICU interventions such as broad-spectrum antibiotic use and parenteral nutrition (19, 20).

Baseline clinical data of the children were collected through structured questionnaires and the Hospital Information System (HIS). The information included demographic data (age, sex, weight, height), perinatal history (mode of delivery, birth weight), feeding history, history of prior antibiotic and microecological modulator use, comorbid diagnoses, and key laboratory indicators at admission (such as C-reactive protein, procalcitonin, white blood cell count, platelet count, creatinine, bilirubin, and arterial blood gas parameters), as well as the physiological parameters required to calculate the pediatric Sequential Organ Failure Assessment (pSOFA) score.

### 2.5 Gene sequencing and bioinformatics analysis

Total genomic DNA was extracted from fecal samples using the cetyltrimethylammonium bromide (CTAB) method. The V3-V4 hypervariable region of the bacterial 16S rRNA gene was amplified using barcode-tagged specific primers. The forward primer was 341F (5′-CCTAYGGGRBGCASCAG-3′) and the reverse primer was 806R (5′-GGACTACNNGGGTATCTAAT-3′). After purification and quantification, the PCR products were subjected to paired-end sequencing (2×250 bp) on an Illumina NovaSeq 6000 platform (Novogene, Beijing, China).

The raw sequencing reads were processed using the QIIME2 platform (version 2022.8). First, the q2-demux plugin was used for demultiplexing and initial quality assessment. Subsequently, the DADA2 pipeline was applied for quality control, denoising, and chimera removal, resulting in high-quality Amplicon Sequence Variants (ASVs). Taxonomic annotation of representative ASV sequences from domain to species level was performed using the q2-feature-classifier plugin against the SILVA 138.1 reference database. To ensure fair diversity comparisons, all samples were rarefied to 45,605 sequences per sample.

Based on the generated ASV table, the following analyses were performed:

① Alpha Diversity: The Shannon index, Simpson index, Pielou’s evenness index, and Dominance index were calculated to assess species diversity and evenness within individual samples.
② Beta Diversity: Principal Coordinates Analysis (PCoA) based on a Weighted Unifrac distance matrix was performed to visualize differences between samples. Permutational multivariate analysis of variance (Adonis) was used to statistically test the overall differences in community structure between groups.
③ Taxonomic Composition Analysis: The relative abundances of taxa at different classification levels (phylum, family, genus) were calculated and compared between groups.
④ Differential Taxon Identification: Linear discriminant analysis effect size (LEfSe) was used to identify microbial taxa with significant abundance differences between the case and control groups. Prior to analysis, taxa with a mean relative abundance below 0.1% across all samples were excluded to reduce background noise. LEfSe employs a three-step process: first, the Kruskal–Wallis test (α = 0.05) detects features with differential abundance between groups; second, pairwise Wilcoxon rank-sum tests assess biological consistency; and third, linear discriminant analysis (LDA) estimates the effect size of each differentially abundant taxon. An LDA score threshold of > 2.0 was considered indicative of a discriminant biomarker. To further validate robustness, we performed an additional differential abundance analysis using the Wilcoxon rank-sum test with Benjamini–Hochberg false discovery rate (FDR) correction (Q < 0.05). Only taxa consistently identified by both LEfSe (LDA > 2) and the FDR-corrected Wilcoxon test were retained as robust microbial signatures for subsequent interpretation. The raw sequence data have been deposited in the Genome Sequence Archive (GSA) (21, 22).

### 2.6 Statistical analysis

Continuous variables are presented as mean ± standard deviation if normally distributed, with between-group comparisons performed using independent samples t-tests. For non-normally distributed continuous variables, data are presented as median (interquartile range), and the Mann-Whitney U test was used for comparisons. Categorical variables are expressed as frequency (percentage), with between-group comparisons conducted using the chi-square test or Fisher’s exact test, as appropriate. All hypothesis tests were two-tailed, and a P-value < 0.05 was considered statistically significant. Statistical analysis of baseline and clinical data was performed using IBM SPSS Statistics version 27.0. For microbiome data, alpha diversity indices were compared using the Mann-Whitney U test, while beta diversity analysis and LEfSe were performed within the QIIME2 and R software (version 4.2.0) environments. To assess the effectiveness of our matching strategy in controlling for antibiotic exposure, we compared antibiotic-related variables between the two groups using the chi-square test (for categorical variables) and the Mann-Whitney U test (for continuous variables). As shown in **Table 1**, there were no significant differences between the groups in the proportion of patients receiving antibiotics, the number of antibiotic classes, or the cumulative days of use prior to sampling (P > 0.05 for all), confirming that the two groups were well-balanced regarding this major confounder.

**Table 1:** Baseline characteristics of the study participants.

Characteristics	Case Group (n=50)	Control Group (n=50)	*P*-value
Demographics			
Male*	30 (60.0)	32 (64.0)	0.680
Age (months)**	7.5 (2.0, 27.3)	6.5 (2.0, 18.8)	0.529
BMI***	15.62 ± 2.59	15.18 ± 2.62	0.395
Birth & Feeding History			
Birth weight (kg)**	3.03 (2.62, 3.36)	3.20 (2.64, 3.50)	0.301
Vaginal delivery*	32 (64.0)	30 (60.0)	0.680
Exclusive breastfeeding (first 6 month), *	26 (52.0)	23 (46.0)	0.360
First complementary feeding age**	6.00 (5.75, 6.00)	6.00 (5.00, 6.00)	0.452
Age at first antibiotic use (year)*			
Never-use	14 (28.00)	16 (32.00)	0.487
<1	18 (36.00)	23 (46.00)	
1~2	13 (26.00)	8 (16.00)	
2~3	5 (10.00)	3 (6.00)	
Age at first probioticsuse (year)*			
Never-use	22 (44.00)	23 (46.00)	0.860
<1	17 (34.00)	17 (34.00)	
1~2	7 (14.00)	8 (16.00)	
2~3	4 (8.00)	2 (4.00)	
Antibiotic Exposure (within 1 month prior to sampling)			
Any antibiotic use*	49 (98.0)	47 (94.0)	0.307
Number of antibiotic classes**	2.0 (1.8, 3.3)	2.0 (1.0, 3.0)	0.210
Cumulative days of use**	3.0 (1.0, 5.0)	3.5 (2.0, 6.0)	0.370
Clinical data			
Pediatric Early Warning Score **	2.00 (1.00, 6.00)	2.00 (1.00, 5.25)	0.826
C-reactive protein (mg/L)**	18.55 (5.33, 44.93)	15.95 (5.51, 35.62)	0.717
White Blood Cell (10^9^/L)**	11.23 (7.05, 15.63)	8.87 (6.78, 13.20)	0.156
ICU length of stay**	13.50 (8.00, 22.00)	10.00 (7.00, 18.50)	0.192
ICU mortality*	3 (6.00)	1 (2.00)	0.307

*, n (%); **, median (IQR); ***, (± S); The case and control groups were successfully matched, with no statistically significant differences in demographic characteristics, perinatal history, or antibiotic exposure parameters (proportion exposed, number of classes, and cumulative days of use), indicating that the two groups were highly comparable and that the potential confounding effects of antibiotics were minimized by design.

## 3 Results

### 3.1 Characteristics of the study population

A total of 100 participants were ultimately included in this study, comprising 50 participants with severe pneumonia combined with sepsis (case group) and 50 participants with severe pneumonia alone (control group). There were no statistically significant differences between the two groups in key demographic characteristics, perinatal history, feeding history, or antibiotic exposure prior to admission. Specifically, the two groups were highly comparable in terms of age in months, sex ratio, birth weight, mode of delivery, feeding patterns within the first six months, as well as the rate, types, and cumulative duration of antibiotic use within one month before sample collection. This indicates that the case-control matching strategy was successful and effectively controlled for these potential confounding factors (**Table 1**).

### 3.2 Overview of sequencing data

**Figure 1:**
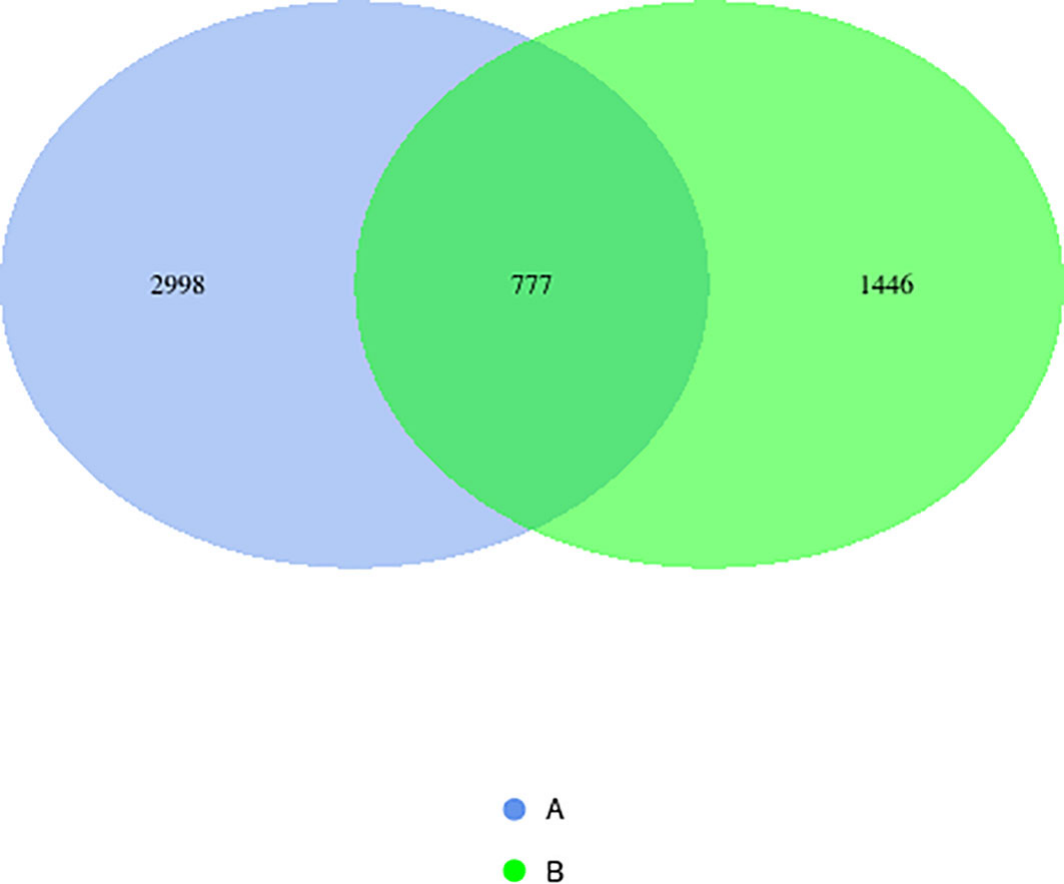
Venn diagram of ASV annotation between two groups.

16S rRNA gene sequencing was performed on 100 fecal samples. After quality control and rarefaction (to 45,605 sequences per sample), a total of 5,221 high-quality amplicon sequence variants (ASVs) were obtained. Taxonomic annotation covered all levels from kingdom to species (kingdom: 99.9%, phylum: 99.8%, class: 99.6%, order: 99.2%, family: 99.0%, genus: 96.3%, species: 18.0%). The Venn diagram showed that the case group and the control group possessed 2,998 and 1,446 unique ASVs, respectively, with 777 ASVs shared between the two groups, indicating that the microbial communities of the two groups shared a common foundation while also exhibiting distinct compositional specificity (**Figure 1**).

### 3.3 Alpha diversity analysis

**Figure 2:**
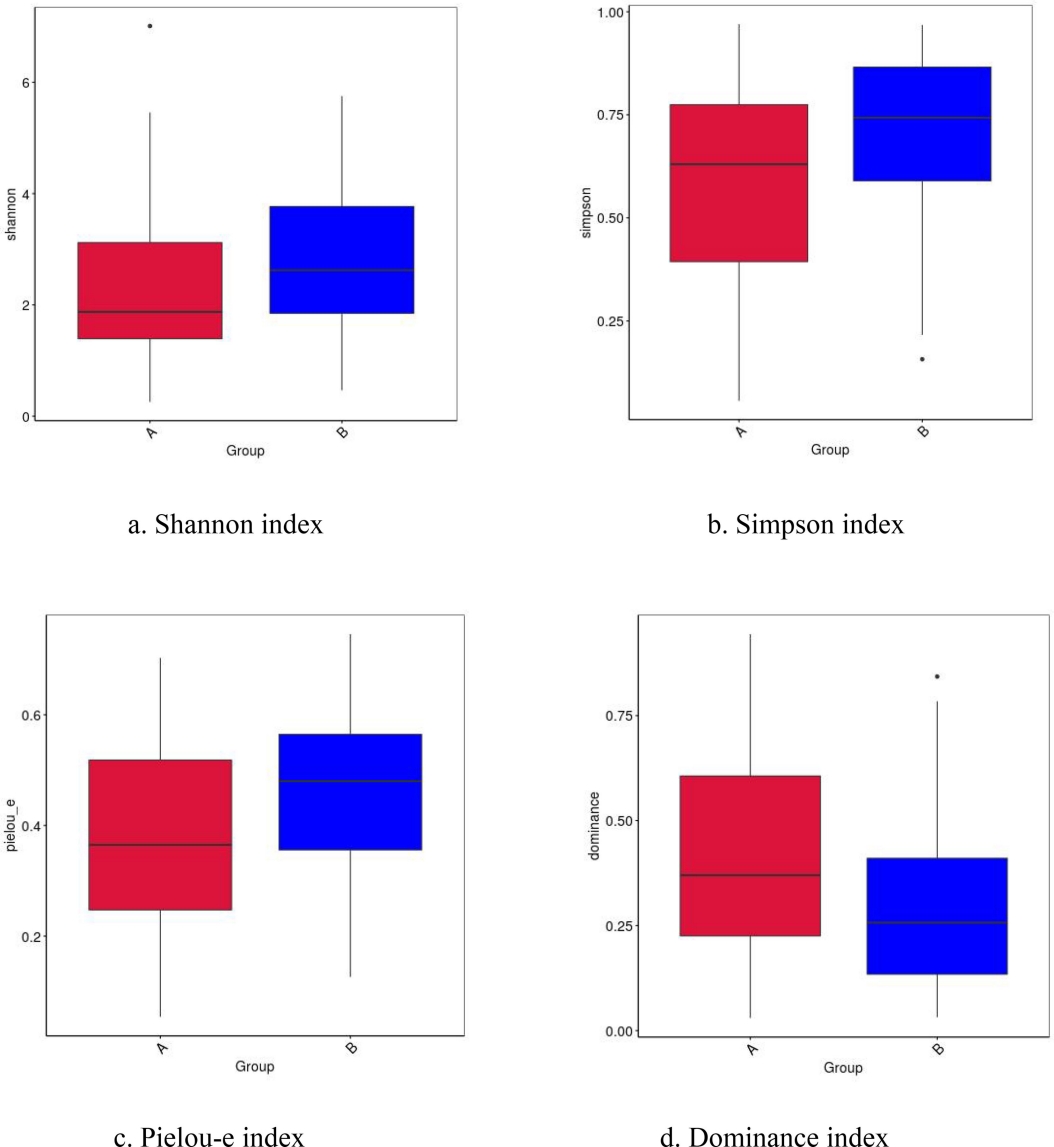
Box plot of alpha diversity differences. The horizontal axis represents the group classification, while the vertical axis corresponds to the Alpha diversity index. 'A' indicates the case group, and 'B' indicates the control group.

The alpha diversity of the intestinal microbiota in the case group was significantly lower than that in the control group (**Figure 2**). Specifically, both the Shannon index (2.30 ± 1.50 vs. 2.83 ± 1.36, P = 0.027) and the Simpson index (0.58 ± 0.26 vs. 0.70 ± 0.21, P = 0.011) were significantly reduced in the case group. Meanwhile, Pielou’s evenness index was also significantly lower in the case group (0.38 ± 0.19 vs. 0.46 ± 0.16, P = 0.015), whereas the dominance index was significantly higher (0.42 ± 0.26 vs. 0.30 ± 0.21, P = 0.011) (**Table 2**). These results indicate that the intestinal microbiota of participants with sepsis comorbidity not only exhibits reduced species diversity but also demonstrates a more uneven community structure, with a few species dominating, presenting a typical state of dysbiosis.

**Table 2:** Comparison of alpha diversity index between the two groups.

Alpha diversity index	Case group Control group		*P* value
	$\bar{x}\pm s$		
Shannon	2.30 ± 1.50	2.83 ± 1.36	0.027
Simpson	0.58 ± 0.26	0.70 ± 0.21	0.011
Pielou-e	0.38 ± 0.19	0.46 ± 0.16	0.015
Dominance	0.42 ± 0.26	0.30 ± 0.21	0.011

Data are presented as mean ± standard deviation.

### 3.4 Beta diversity analysis

**Figure 3:**
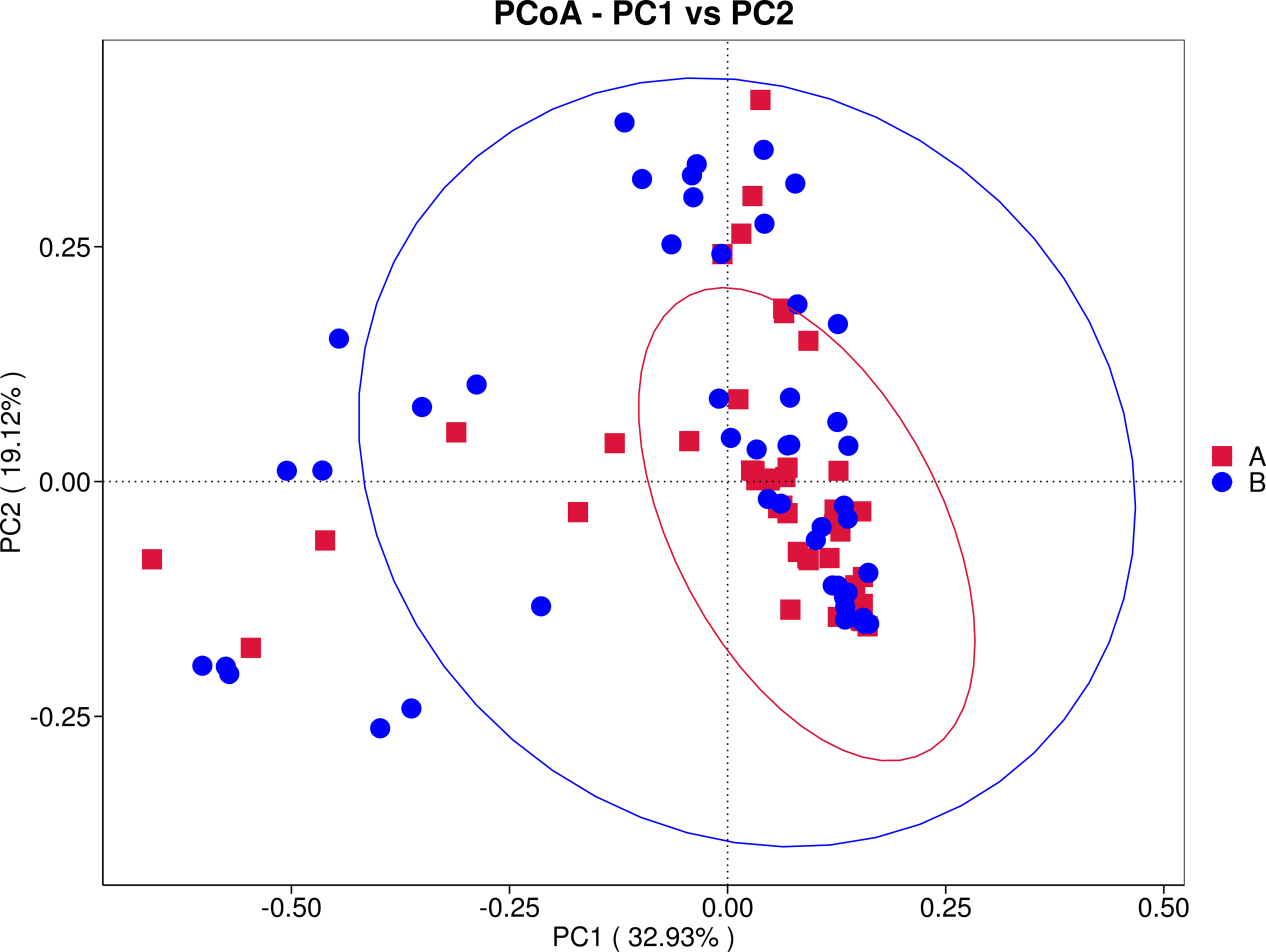
PCoA plot. Red 'A' represents the case group, and blue 'B' represents the control group.

**Figure 4:**
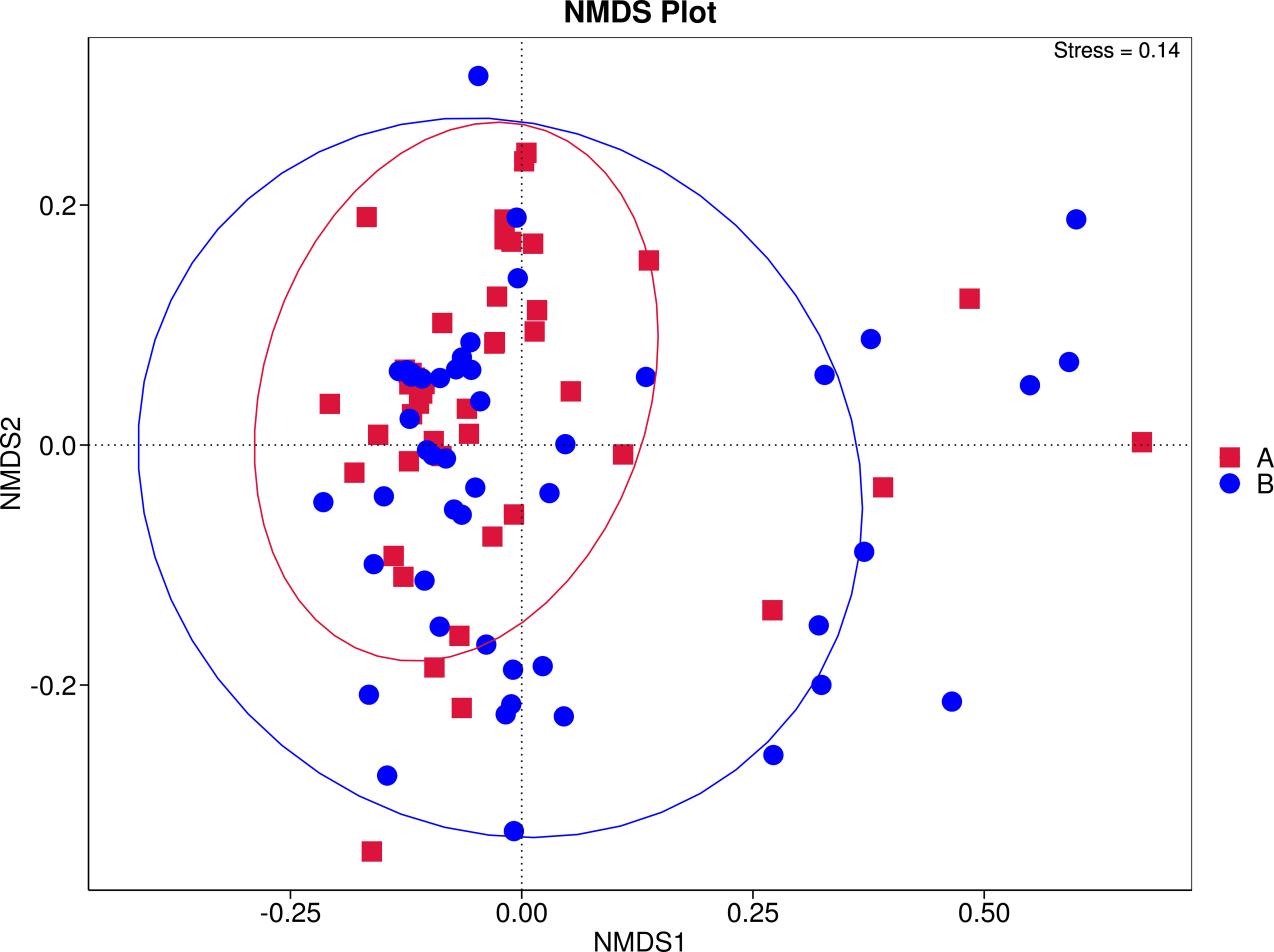
NMDS plot. Samples from the case group and the control group are represented by red ‘A’ and blue ‘B’ markers, respectively.

Beta diversity analysis was performed to assess overall structural differences in the intestinal microbiota between the two groups. Principal Coordinate Analysis (PCoA) based on Weighted Unifrac distance reduced the data to two dimensions (PC1: 32.93%; PC2: 19.12%), revealing that although samples from the case (A) and control (B) groups showed partial clustering, considerable overlap was observed, suggesting any structural differences were subtle (**Figure 3**). Non−metric multidimensional scaling (NMDS; stress = 0.14, indicating good fit) further confirmed this pattern, with visible overlap alongside limited separation (**Figure 4**). These results imply that overall inter−group differences in microbiota composition require further statistical validation. Permutational multivariate analysis of variance (Adonis) further confirmed that there was no statistically significant difference in the overall community structure (Beta diversity) of the intestinal microbiota between the two groups (*R²* = 0.012, *P* = 0.193) (**Table 3**). This suggests that the global architecture of the gut microbiota was largely shaped by the common underlying condition of severe pneumonia and antibiotic exposure. However, this lack of global differentiation does not preclude the existence of significant abundance shifts in specific, key taxa, which were subsequently investigated using differential abundance analysis.

**Table 3:** Adonis analysis of the intestinal microbiota between the two groups.

Groups	*Df*	*SumsOfSqs*	*MeanSqs*	*F.Model*	*R^2^*	*Pr(>F)*
A-B	1(98)	0.491	0.491	1.213	0.012	0.193

Df, degrees of freedom; SumsOfSqs, sums of squares; MeanSqs, mean squares; F.Model, F statistic in ANOVA; R², proportion of variance explained by the grouping factor; Pr, P-value.

### 3.5 Relative abundance analysis

The intestinal microbiota in both groups was primarily composed of four major phyla: Bacillota, Pseudomonadota, Actinomycetota, and Bacteroidota, indicating a shared foundational composition. However, discernible structural differences emerged at finer taxonomic resolutions.

At the family level, significant differences were observed within the top 10 most abundant taxa. Specifically, the relative abundance of Enterobacteriaceae was significantly higher in the sepsis group (18.97% vs. 9.44%, *P* = 0.046), while Lachnospiraceae was significantly lower (2.01% vs. 8.11%, *P* = 0.010). No significant differences were found for the other highly abundant families.

At the species level, among the top 10 species, the sepsis group exhibited significantly lower relative abundances of Unclassified_Megamonas (0.16% vs. 0.99%, *P* = 0.041) and Unclassified_Segatella (0.01% vs. 0.67%, *P* = 0.025). The remaining highly abundant species showed no statistically significant differences between the two groups.

In summary, while the overall microbial architecture was similar, the sepsis group was characterized by a distinct signature marked by an increase in Enterobacteriaceae and decreases in Lachnospiraceae, Unclassified_Megamonas, and Unclassified_Segatella.

### 3.6 Comparative microbiota analysis

**Figure 5:**
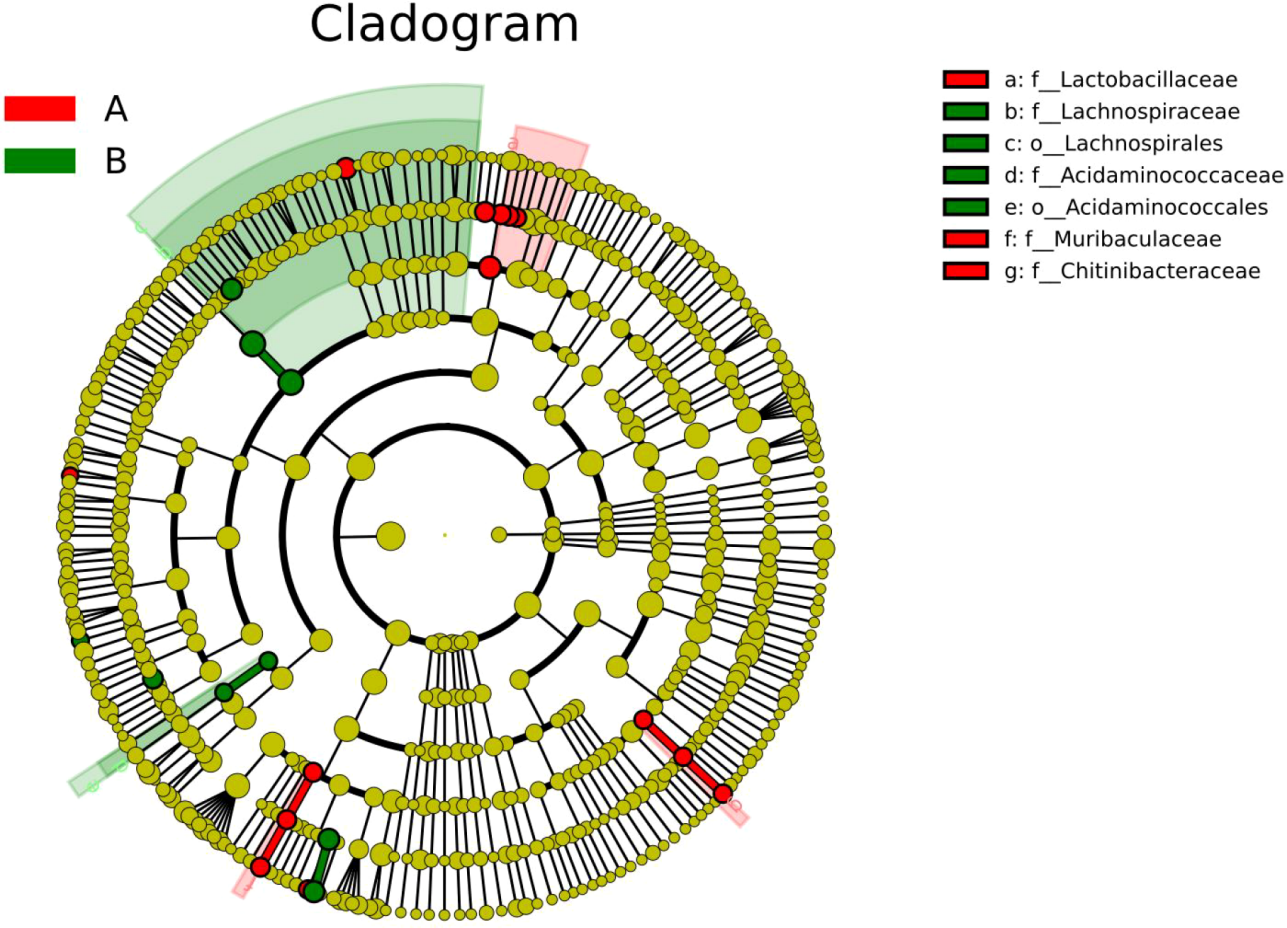
Phylogenetic tree. The concentric ring structure in the figure, arranged from the inside outward, corresponds to taxonomic levels from phylum to genus, respectively. Each node on a ring represents a taxonomic unit at that level, with its diameter proportional to the relative abundance (RA) value. Taxa significantly enriched in the case group are colored in red, while those significantly enriched in the control group are colored in green.

To systematically identify differentially abundant taxa and determine dominant biomarkers between the groups, LEfSe (Linear Discriminant Analysis Effect Size) was performed (LDA score > 2)(**Figure 5**). The analysis revealed distinct microbial signatures.

In the sepsis group (case group), the most discriminative biomarkers were Lactobacillaceae at the family level (LDA = 3.62), Pediococcus at the genus level (LDA = 3.15), and Clostridium butyricum at the species level (LDA = 3.50).

Conversely, the control group was characterized by enrichment of Lachnospiraceae at the family level (LDA = 4.49), Segatella at the genus level (LDA = 3.57), and Unclassified_Segatella at the species level (LDA = 3.57).

To further evaluate the discriminant taxa identified by LEfSe, we performed an additional differential abundance analysis using the Wilcoxon rank-sum test. The results showed that Lachnospiraceae (family), Segatella (genus), Clostridium butyricum (species), and Lactobacillaceae (family) remained significantly different between the two groups (P < 0.05), consistent with the LEfSe findings. This consistency supports the potential of these microbial features as robust signatures associated with sepsis in infants and young children with severe pneumonia.

These results clearly delineate group-specific microbial biomarkers across taxonomic levels, with Lactobacillaceae/Lachnospiraceae and Clostridium butyricum/Unclassified_Segatella representing the most prominent discriminative features at the family and species levels, respectively.

## 4 Discussion

### 4.1 Global similarity vs. local shifts: interpreting the beta diversity findings

An intriguing observation of our study is the discordance between the lack of significant global beta diversity difference (Adonis P = 0.193) and the presence of significant alterations in specific taxa. We interpret this finding as follows. First, the stringent matching for age and antibiotic exposure, along with the shared diagnosis of severe pneumonia, likely created a common baseline dysbiotic state in both groups (23, 24). The profound effects of critical illness and antibiotics on the gut ecosystem may have overshadowed any sepsis-specific effect at the global community level. By effectively accounting for confounders, our control group allows the residual differences—such as Enterobacteriaceae enrichment and Lachnospiraceae depletion—to be interpreted as the specific microbial signature associated with sepsis progression. Second, beta diversity analyses are global tests that may lack sensitivity to detect shifts confined to a subset of keystone taxa (25, 26). In our study, the sepsis-specific ‘signal’ appears restricted to a relatively small number of functionally important bacteria. These changes, while biologically significant and detectable by LEfSe, were not substantial enough to drive a statistically significant shift in overall community distance metrics. Thus, our findings suggest that sepsis-associated dysbiosis is characterized by specific ecological imbalances within a broadly similar community structure, rather than a complete community overhaul. This underscores the value of combining global and taxon-specific analytical approaches to fully capture the complex nature of dysbiosis.

### 4.2 Summary of major findings

In this well-matched case-control study of infants and young children with severe pneumonia, we demonstrated that those complicated by sepsis exhibited a distinct gut microbial profile compared to those without sepsis. Key alterations included a significant reduction in alpha diversity, expansion of the potential pathobiont Enterobacteriaceae, depletion of beneficial or functional taxa such as Lachnospiraceae and Segatella, and a unique biomarker spectrum identified by LEfSe analysis. These findings suggest that the presence of sepsis is associated with a specific dysbiotic pattern that accompanies the already altered gut microbiome of critically ill children (24, 27, 28).

### 4.3 Significance of diversity loss

The observed decrease in alpha diversity indices (Shannon, Simpson, Pielou’s evenness) along with increased Dominance in the sepsis group aligns with the classic hallmark of gut ecological collapse in critical illness (7, 28). Similar diversity loss has been consistently reported in adult and pediatric sepsis cohorts, reflecting a compromised microbial ecosystem under severe physiological stress (1, 23). This depleted, uneven community structure may be indicative of reduced functional redundancy and resilience, which could potentially compromise the gut’s ability to resist pathogen invasion or recover homeostasis, and might contribute to systemic inflammation and organ dysfunction (28, 29).

### 4.4 Expansion of a potential pathobiont: Enterobacteriaceae

The near doubling of Enterobacteriaceae relative abundance in septic patients may have important pathological implications. The observed expansion of Enterobacteriaceae, a family encompassing many LPS-producing opportunistic pathogens, raises the hypothesis that it may contribute to sepsis pathogenesis. Based on their known functional potential, it is plausible that an increased abundance of these bacteria could compromise intestinal barrier integrity, and that their components (e.g., LPS) or metabolites might translocate into systemic circulation, potentially triggering or amplifying pro-inflammatory cascades. However, as our study provides only taxonomic data, direct evidence for increased LPS translocation or barrier dysfunction is lacking. This proposed mechanism remains a hypothesis that requires validation through future functional analyses, such as measurement of plasma LPS or metabolomic studies (30, 31). Previous studies have associated Enterobacteriaceae enrichment with worse clinical outcomes and sustained inflammation in sepsis, supporting its role as both a marker and mediator of disease severity (32, 33).

### 4.5 Depletion of beneficial/functional taxa: Lachnospiraceae and Segatella

The reduction of Lachnospiraceae, a key butyrate-producing family, may have multifaceted detrimental effects. Butyrate serves as the primary energy source for colonocytes, maintains epithelial barrier function, and exerts anti-inflammatory and immunomodulatory activities. Its shortage might contribute to epithelial energy deficit, compromised tight junction integrity, and dysregulated mucosal immunity, collectively potentially exacerbating gut leakiness and systemic inflammatory burden in sepsis (34, 35).

Similarly, the depletion of Segatella (and Megamonas), both involved in carbohydrate fermentation and metabolic networking, suggests a broader disruption of microbial metabolic capacity. This may reflect a loss of functional modules essential for nutrient processing, energy harvest, and microbial community stability, further compromising host-microbe mutualism under septic stress (36, 37).

### 4.6 Differential taxa as potential biomarkers

LEfSe analysis revealed a biomarker signature that may assist in risk stratification. Intriguingly, traditionally considered “probiotic” taxa such as Lactobacillaceae and Clostridium butyricum were enriched in the sepsis group. This may represent a compensatory response aimed at countering dysbiosis or, alternatively, opportunistic overgrowth of certain acid-tolerant or inflammatory-resistant strains within a severely disrupted ecosystem. Conversely, the control-enriched taxa, particularly Lachnospiraceae and Segatella, could serve as negative biomarkers; their higher abundance or proportional representation might indicate a more preserved gut microbiome, potentially predicting a lower risk of sepsis progression in children with severe pneumonia (8, 38).

### 4.7 Clinical implications and insights

The distinct dysbiotic pattern associated with pediatric sepsis holds direct implications for clinical practice. Firstly, the microbial signature (e.g., elevated Enterobacteriaceae, depleted Lachnospiraceae) could aid in the early identification of high-risk patients among those with severe pneumonia, prompting closer monitoring and aggressive supportive care (7, 28). Secondly, these findings reinforce the importance of judicious antibiotic stewardship in the PICU, as broad-spectrum agents may exacerbate the loss of beneficial taxa and promote pathogen expansion (23, 39). Thirdly, the depletion of key functional bacteria supports the rationale for targeted microbial or postbiotic interventions (e.g., butyrate producers, selective prebiotics) to restore barrier and metabolic functions, though timing and strain selection require careful study in acute sepsis (40, 41). For nursing and nutritional care, emphasis on early enteral feeding with fiber-rich or specially formulated diets may help support residual beneficial microbes and mitigate dysbiosis (5, 42). Overall, integrating gut microbiome assessment into the management framework of critically ill children could pave the way for more personalized and ecologically informed therapeutic strategies (1).

## 5 Limitations and future directions

Several limitations should be acknowledged. First, the matched case−control design precludes causal inference; while we identified significant associations between gut microbiota dysbiosis and sepsis, the temporal direction—whether dysbiosis predisposes to sepsis or results from it—cannot be determined.

Second, despite rigorous 1:1 matching that successfully balanced key antibiotic metrics (number of classes, cumulative days) between groups (**Table 1**), antibiotic exposure remains a potent confounder in critically ill populations. Residual confounding from nuanced factors (e.g., dosing regimens, specific drug combinations) cannot be entirely ruled out, and the fact that only one child in the sepsis group and three in the control group remained antibiotic−naïve at sampling precluded meaningful stratified analysis to isolate sepsis−specific effects.

Third, this study relied on 16S rRNA amplicon sequencing, which provides taxonomic but not functional metagenomic information. Consequently, mechanistic interpretations—such as the role of Enterobacteriaceae in endotoxin production or Lachnospiraceae depletion in butyrate deficiency—are inferred from known properties of these taxa and remain hypothetical, warranting validation through shotgun metagenomics and metabolomics.

Fourth, although our initial power calculation targeted 53 participants per group to account for 15% attrition, the final analysis included 50 per group (actual attrition 5.7%). A post−hoc power analysis based on the observed effect size for the primary outcome (Shannon index, Cohen’s d ≈ 0.37) confirmed >80% power at α = 0.05, indicating that this slight reduction did not materially affect the main conclusions. Nevertheless, larger multi−center longitudinal studies integrating metagenomics and metabolomics are warranted to validate these microbial signatures, elucidate causal pathways, and inform microbiome−based strategies for early risk stratification in pediatric sepsis.

## 6 Conclusion

This well-matched case-control study demonstrates that infants and young children with severe pneumonia complicated by sepsis exhibit a distinct gut microbial dysbiosis, characterized by significantly reduced alpha diversity, expansion of the potential pathobiont Enterobacteriaceae, and depletion of beneficial or functional taxa such as Lachnospiraceae, Segatella, and Megamonas. LEfSe analysis further identified a set of discriminant microbial biomarkers.

These specific microbial alterations involve taxa associated with key pathophysiological processes in sepsis, such as potential endotoxin release (e.g., Enterobacteriaceae) and short-chain fatty acid production (e.g., Lachnospiraceae), offering potential biomarkers for early risk stratification. However, the proposed functional consequences of this dysbiosis remain hypotheses. Although the cross-sectional design precludes causal inference and the findings are subject to limitations inherent to a single-center study with moderate sample size and pervasive antibiotic exposure, the results strongly suggest that gut microbiota structure is significantly associated with the pathoecological landscape in sepsis. Future multi-center, large-scale longitudinal studies employing metagenomic and metabolomic approaches are warranted to validate the predictive value of these microbial signatures, elucidate underlying functional mechanisms, and inform the development of early-warning strategies and microbiome-based adjunctive therapies for sepsis.

## Data Availability

The raw sequence data reported in this paper have been deposited in the Genome Sequence Archive (Genomics, Proteomics & Bioinformatics 2025) in National Genomics Data Center (Nucleic Acids Res 2025), China National Center for Bioinformation / Beijing Institute of Genomics, Chinese Academy of Sciences (GSA: CRA040258) that are publicly accessible at https://ngdc.cncb.ac.cn/gsa. [41,42]
